# Correction: Removal of long-acting reversible contraceptive methods and quality of care in Dar es Salaam, Tanzania: Client and provider perspectives from a secondary analysis of cross-sectional survey data from a randomized controlled trial

**DOI:** 10.1371/journal.pgph.0003494

**Published:** 2024-07-05

**Authors:** The PLOS Global Public Health Staff

## Notice of Republication

An incorrect version of Figure 1 was published in error. The publisher apologizes for this error. This article was republished on June 25, 2024 to correct for this error. Please download this article again to view the correct version.

## Supporting information

S1 File Originally published uncorrected article. (PDF)

S2 File Republished corrected article. (PDF)
