# Supplementary material for: Removal of long-acting reversible contraceptive methods and quality of care in Dar es Salaam, Tanzania: Client and provider perspectives from a secondary analysis of cross-sectional survey data from a randomized controlled trial
Source: PLOS Glob Public Health. 2024 Jan 23;4(1):e0002810. doi: 10.1371/journal.pgph.0002810 (PMC10805313; doi:10.1371/journal.pgph.0002810)
Supplement: S1 Table — (DOCX) [file pgph.0002810.s002.docx]

S1 Table: Items in autonomy and respect subscale of a validated *person-centered family planning scale*

| **Original question** | **Study version of question** |
| --- | --- |
| *Included in paper scale* |  |
| Did the doctors, nurses, or other staff at the facility treat you with respect? | Did the provider you saw for family planning treat you with disrespect?* |
| Did the doctors, nurses, and other staff at the facility treat you in a friendly manner? | Did the provider you saw for family planning treat you in an unfriendly manner?* |
| Did the doctors, nurses, and other staff at the facility show that they cared about you? | Did you feel the provider you saw for family planning cares about you as a person? |
| Did the doctors and nurses at the facility talk to you about how you were feeling? | Did the provider you saw for family planning talk to you about how you were feeling? |
| Did you feel you could ask the doctors, nurses or other staff at the facility any questions you had? | Did you feel you could ask the provider you saw for family planning at the facility any questions you had? |
| Did you feel the doctors and nurses paid attention to you during your stay in the facility? | Did you feel the provider you saw for family planning paid attention to you during your stay in the facility? |
| Did you feel you could completely trust the doctors, nurses or other staff at the facility with regards to your care? | Did you feel you could completely trust the provider you saw for family planning with regards to your care? |
| Were you allowed to have someone you wanted to stay with you during your visit? | Were you allowed to have someone you wanted to stay with you during your visit? |
| Did you feel like the doctors, nurses or other staff at the facility involved you in decisions about your FP Choice. | Did you feel like the provider you saw for family planning involved you in decisions about your FP choice? |
| Did the doctors, nurses or other staff at the facility speak to you in a language you could understand? | Did you feel the provider you saw for family planning at the facility clearly explained things to you? |
| *Not included* |  |
| How did you feel about the amount of time you waited? | *-* |
| Did the doctors, nurses, or other health care providers call you by your name? | *-* |
| Did you feel the doctors, nurses or other staff at the facility took the best care of you? |  |
| During your time in this clinic did the doctors, nurses, or other health care providers introduce themselves to you when they first came to see you? | During your time in the health facility did the provider you saw for family planning introduce themselves to you when they first came to see you? (*Included as a binary version of this question which is not included in the final scale for this reason)* |
