# Supplementary material for: Removal of long-acting reversible contraceptive methods and quality of care in Dar es Salaam, Tanzania: Client and provider perspectives from a secondary analysis of cross-sectional survey data from a randomized controlled trial
Source: PLOS Glob Public Health. 2024 Jan 23;4(1):e0002810. doi: 10.1371/journal.pgph.0002810 (PMC10805313; doi:10.1371/journal.pgph.0002810)
Supplement: S2 Table — (DOCX) [file pgph.0002810.s003.docx]

S2 Table: Association between LARC removal and person-centered autonomous and respectful care: Model results (n= 2,235)

|  | **Model 1** | **Model 2** | **Model 3** | **Model 4** |
| --- | --- | --- | --- | --- |
|  | Main effects unadjusted | Main effects adjusted | Interaction between LARC removed and treatment | Interaction between LARC removed and treatment adjusted |
| LARC removed | 0.08** (0.03 - 0.13) | 0.07** (0.02 - 0.11) | 0.03 (-0.03 - 0.09) | 0.03 (-0.03 - 0.09) |
| Treatment | 0.03 (-0.09 - 0.15) | 0.04 (-0.08 - 0.16) | -0.07 (-0.22 - 0.08) | -0.05 (-0.20 - 0.10) |
| LARC removed* Treatment |  |  | 0.11* (0.02 - 0.21) | 0.10 (0.00 - 0.19) |
| Client age |  |  |  |  |
| ≤19 |  | Ref |  | Ref |
| 20-24 |  | 0.05 (-0.04 - 0.14) |  | 0.05 (-0.05 - 0.14) |
| >24 |  | 0.11* (0.01 - 0.20) |  | 0.10* (0.01 - 0.19) |
| Married |  | 0.05** (0.02 - 0.08) |  | 0.05** (0.02 - 0.08) |
| Parity |  |  |  |  |
| No children |  | Ref |  | Ref |
| 1 child |  | -0.06 (-0.15 - 0.02) |  | -0.06 (-0.14 - 0.02) |
| 2 children |  | -0.08 (-0.17 - 0.01) |  | -0.08 (-0.16 - 0.01) |
| 3+ children |  | -0.09 (-0.18 - 0.00) |  | -0.09 (-0.17 - 0.00) |
| Secondary plus education |  | 0.03* (0.00 - 0.06) |  | 0.03* (0.00 - 0.06) |
| Perceived socioeconomic status |  |  |  |  |
| Lowest |  | Ref |  | Ref |
| Middle |  | -0.02 (-0.05 - 0.02) |  | -0.02 (-0.05 - 0.02) |
| Highest |  | 0.01 (-0.07 - 0.09) |  | 0.00 (-0.07 - 0.09) |
| Accompanied to visit |  | -0.08*** (-0.11 - -0.05) |  | -0.08*** (-0.11 - -0.04) |

Estimated with linear mixed effects models with random intercepts at the facility-level.

* p<.05 ** p<.01 *** p<.001
