# Supplementary material for: Correction: Removal of long-acting reversible contraceptive methods and quality of care in Dar es Salaam, Tanzania: Client and provider perspectives from a secondary analysis of cross-sectional survey data from a randomized controlled trial
Source: PLOS Glob Public Health. 2024 Jul 5;4(7):e0003494. doi: 10.1371/journal.pgph.0003494 (PMC11226037; doi:10.1371/journal.pgph.0003494)
Supplement: S1 File — (PDF) [file pgph.0003494.s001.pdf]

PLOS GLOBAL PUBLIC HEALTH

RESEARCH ARTICLE

Removal of long-acting reversible contraceptive methods and quality of care in Dar es Salaam, Tanzania: Client and provider perspectives from a secondary analysis of cross-sectional survey data from a randomized controlled trial

Alexandra Wollum[1,2]*, **Corrina Moucheraud**[3], **Amon Sabasaba**[4], **Jessica D. Gipson**[1,2]

1 Department of Community Health Sciences, Fielding School of Public Health, University of California, Los Angeles, Los Angeles, CA, United States of America, **2** The UCLA Bixby Center on Population and Reproductive Health, Los Angeles, California, United States of America, **3** Department of Public Health Policy and Management, School of Global Public Health, New York University, New York City, New York, United States of America, **4** Health for a Prosperous Nation (H-PON), Dar es Salaam, Tanzania

* awollum@ucla.edu

Abstract

Access to removal of long-acting reversible contraception (LARCs) (e.g., implants and intrauterine devices (IUDs)) is an essential part of contraceptive care. We conducted a secondary analysis of cross-sectional survey data from a randomized controlled trial. We analyzed 5,930 client surveys and 259 provider surveys from 73 public sector facilities in Tanzania to examine the receipt of desired LARC removal services among clients and the association between receipt of desired LARC removal and person-centered care. We used provider survey data to contextualize these findings, describing provider attitudes and training related to LARC removals. All facilities took part in a larger randomized controlled trial to assess the Beyond Bias intervention, a provider-focused intervention to reduce provider bias on the basis of age, marital status, and parity. Thirteen percent of clients did not receive a desired LARC removal during their visit. Clients who were young, had lower perceived socioeconomic status, and visited facilities that did not take part in the Beyond Bias intervention were less likely to receive a desired removal. Clients who received a desired LARC removal reported higher levels of person-centered care ($\beta = .07$, CI: .02 - .11, $p = < .01$). Half of providers reported not being comfortable removing a LARC before its expiration (51%) or if they disagreed with the client's decision (49%). Attention is needed to ensure clients can get their LARCs removed when they want to ensure patient-centered care and protect client autonomy and rights. Interventions like the Beyond Bias intervention, may work to address provider-imposed barriers to LARC removals.

OPEN ACCESS

Citation: Wollum A, Moucheraud C, Sabasaba A, Gipson JD (2024) Removal of long-acting reversible contraceptive methods and quality of care in Dar es Salaam, Tanzania: Client and provider perspectives from a secondary analysis of cross-sectional survey data from a randomized controlled trial. PLOS Glob Public Health 4(1): e0002810. https://doi.org/10.1371/journal.pgph.0002810

Editor: Ifunanya Clara Agu, Health Policy Research Group, University of Nigeria, NIGERIA

Received: July 22, 2023

Accepted: December 20, 2023

Published: January 23, 2024

Copyright: © 2024 Wollum et al. This is an open access article distributed under the terms of the Creative Commons Attribution License, which permits unrestricted use, distribution, and reproduction in any medium, provided the original author and source are credited.

Data Availability Statement: The Beyond Bias datasets are available on the Harvard Dataverse. The client exit data are available at https://doi.org/10.7910/DVN/KCKV3R. The provider survey data are available at https://doi.org/10.7910/DVN/CAEVFV.

Funding: Research reported in this paper was supported by the Eunice Kennedy Shriver National

Institute Of Child Health & Human Development of the National Institutes of Health under Award Number F31HD111291 (AW). The content is solely the responsibility of the authors and does not necessarily represent the official views of the National Institutes of Health. The funders had no role in study design, data collection and analysis, decision to publish, or preparation of the manuscript.

Competing interests: The authors have declared that no competing interests exist.

1. Introduction

The use of long-acting reversible contraceptives (LARCs; intrauterine devices or IUDs and implants), and in particular implants, has increased dramatically in the past decade in sub-Saharan Africa [1]. From 2014 to 2020, implant procurement more than doubled, so that in 2016 and 2017 implants were among the most utilized contraceptive method in many sub-Saharan African countries [1, 2]. These increases reflect a growing enthusiasm for LARC methods globally; LARCs are often prioritized in contraceptive counseling and have been promoted to address rates of unintended pregnancy due to their high efficacy rate, long duration of use, and because they require no action following insertion on the part of the user [3–5].

Unlike other reversible contraceptive methods, it is recommended that LARCs are removed by a clinician. This reliance on clinician involvement creates an opportunity for providers to exert influence when people seek to get their LARC method removed for the purposes of discontinuing, switching, or continuing the method once expired.

An estimated 4.9 million people sought implant removals across 69 low- and middle-income countries (LMICs) in 2018, an increase of ~125% from 2015 [6]. (No comparable estimate is available for IUDs.) Despite this increase, there has been little attention to LARC removal in LMICs, prompting researchers and advocates to identify LARC removals as an overlooked aspect of contraceptive care, with training and availability of services for removals lagging behind insertions [6]. The limited existing literature on LARC removals from LMICs points to barriers including long wait times, associated costs, provider refusal and unavailability, and provider training [2, 7–12]. Providers can act as gatekeepers to LARC removal, encouraging clients to continue use of the method or refusing to provide the service altogether, reflecting what has also been documented in the U.S. [12–14]. Such barriers may interfere with clients' right to stop using contraception when they want, or to use the method they choose. LARC removal upon user request has been clearly articulated as a component of high quality, person-centered care and essential to uphold the tenets of reproductive justice, "the right to maintain bodily autonomy, have children, not have children and parent children in safe and sustainable communities" [15–18]. Person-centered care is defined as care that is respectful of and responsive to clients' preferences and needs and driven by client values [19, 20] and in the context of LARC removals, prioritizes the client's desire to have a LARC removed at any and for whatever reasons they deem important (e.g., side effects, desire to switch or discontinue contraception altogether, desire to become pregnant etc.).

In this paper, we utilize three sources of data from Tanzania, a country in Eastern Africa, to examine LARC removals from both a provider and client perspective. We examine the receipt of desired LARC removal services among clients and the association between receipt of desired LARC removal and person-centered care. We also use provider survey data to contextualize these findings, describing provider attitudes and training related to LARC removals.

2. Materials and methods

2.1 Study setting

This study involved clients and a sample of providers from 73 public health facilities in Dar es Salaam, Tanzania. In Tanzania, the use of implants and IUDs has increased by 1240% and 80% respectively since 2004. Tanzania has received large implant commodity distributions, in part fueled by a donation of over 4.1 million implants by global donors between 2013 and 2017 [1, 21]. Furthermore, Tanzania has set explicit targets for increasing LARC use—aiming for a 20% and 13% increase in implant and IUD use respectively between 2019 and 2023, increasing the structural emphasis on LARC uptake [22].

2.2 Parent study

This study drew on two sources of data collected as part of the Beyond Bias study, a mixed-methods evaluation of a facility-based intervention to reduce provider bias on the basis of client age, marital status, and parity in contraceptive care in Tanzania [23]. The study was conducted between September 2020 and August 2021. Seventy-three facilities were included in the study; all were public sector primary care facilities in four districts of Dar es Salaam, Tanzania (Ilala, Kigamboni, Kinodoni, and Temeke). Facilities were located in both urban and periurban settings. Facilities were selected based on holding preexisting relationships with Pathfinder International, the implementing organization of the Beyond Bias intervention. Facilities were randomized 1:1 into the control and intervention arms of the study. There were 36 facilities randomly assigned to the treatment arm and 37 to the control arm. The authors accessed data for the purposes of this analysis from October 2022 to July 2023.

2.3 The intervention

The intervention was a provider-focused program implemented over the course of a year that aimed to support providers to identify and recognize their own biases, build a professional community to implement unbiased care, and support unbiased care through social reinforcement and non-monetary awards. Three programmatic activities were implemented to achieve these aims: (1) a Summit, a story-driven one-day event designed to facilitate dialogue and reflection on provider bias and providers' own behaviors; (2) Connect, an interactive forum for knowledge sharing and learning; and (3) Rewards, a non-financial performance-based incentive to deliver non-biased care. The intervention was developed using a human-centered design approach, which included an assessment of bias and research to identify the most promising intervention design to address the issue [24]. The majority of intervention providers participated in at least some component of the Beyond Bias program (98%). The Beyond Bias intervention did not have any content explicitly focused on LARC removals. For more information on the intervention see Wagner et al. [23].

2.4 Inclusivity in global research

Additional information regarding the ethical, cultural, and scientific considerations specific to inclusivity in global research is included in the S1 Checklist.

2.5 Ethics statement

This is a secondary analysis of deidentified data that was exempted from ethics review by the UCLA IRB. Research participants provided verbal consent to participate in the original study.

2.6 Data sources and study population

a. Client exit surveys. At all 73 facilities, trained enumerators (aged 18–24) conducted exit surveys with clients as they exited the facility after their visit for contraceptive care over the full course of the study period. The approximately 45 enumerators, who had been trained on research ethics, the survey instrument, and study procedures before fielding the survey, visited all facilities two to four times per week to recruit clients to participate in client exit surveys. Surveys were administered by the enumerator who asked questions aloud and entered client responses on a tablet in Kiswahili. Surveys lasted on average 15 minutes. They survey captured client characteristics, and information about their visit including their interactions with providers. Female clients who sought contraceptive care or received contraceptive counseling or care were eligible to participate in the survey. For this analysis, we limited the sample to clients

seeking a LARC removal; clients who came to the facility using an implant and IUD and who did not indicate they were only visiting for advice or for a checkup (22% of the sample).

b.Provider surveys. All contraceptive care providers at enrolled facilities were invited to participate in the survey (82% response rate) in July and August of 2021. Providers from 72 of the 73 facilities were surveyed. Twenty enumerators from a local research firm administered the surveys which captured provider characteristics, attitudes, and self-reported practices in contraceptive care. Enumerators participated in a multi-day training on the survey instrument and data collection procedures. The survey was piloted prior to fielding. Enumerators called providers to schedule appointments to conduct the survey. Enumerators conducted the survey aloud in Kiswahili in person. Surveys lasted on average 45 minutes to an hour.

2.7 Measures

a. Receipt of LARC removal. Clients classified as "received a desired LARC removal" were those who said their IUD or implant was removed, and those who had been using an IUD or implant and received a new method during the visit (as this necessitated removal of their IUD or implant). Clients classified as "did not receive a desired LARC removal" were those who did not receive any services that day, those who explicitly said their IUD or implant had not been removed (sometimes through open-text responses), and those who continued with their LARC and did not receive any method of contraception (as this would have necessitated removal of their IUD or implant).

b. Person-centered care. To measure person-centered care, we used a measure of autonomy and respect reported by the client, one of the two subscales that comprise the validated *person-centered family planning scale (PCFP)* [19]. The autonomy and respect subscale reflects whether the client received care in a manner they found respectful and caring, whether the client felt supported to make informed choices about her care, whether the client trusted and had confidence in her provider, and whether the communication and explanations provided were adequate and understandable. The second subscale of the PCFP assesses the health facility environment. We did not include this subscale as it does not reflect provider-client interactions and is, thus, less relevant to this analysis. The client exit survey instrument included 10 of 14 measures from the validated autonomy and respect subscale (S1 Table); 4 items were excluded because they were not relevant to the evaluation parent study. Items were answered on a four-point scale ("No, never" to "Yes, all of the time"). We took the average across these 10 items to create the measure used in this study (Cronbach's alpha = .73), following the PCFP authors' recommendation [19].

c. Client characteristics. We measured the following client characteristics: client age, relationship status (married, in a relationship not living together, in a relationship living together, and single), parity (no children, 1 child, 2 children, 3+ children), education level (less than secondary, secondary or more), and perceived socioeconomic status (low (step 1–2), medium (step 3–4), and high (step 5–6)). Perceived socioeconomic status was assessed using the question "Imagine six steps, where on the bottom, the first step, stand the poorest people, and on the highest step, the sixth, stand the rich. On which step are you today?" [25]. We also examined whether the client was accompanied to the visit (yes, no) and stated pregnancy intention (desire for a/another pregnancy at all and based on amount of time). Only clients who stated they received services at the facility (either counseling or provision of contraceptive services) were asked about their pregnancy intention.

d. Provider measures. We analyzed measures of provider training in LARC removals, self-reported provision of care, and measures of provider attitudes related to LARC removals using data from the provider survey.

Providers were asked to state their agreement with the following statements to assess their attitudes about early LARC removals: "If a client wants an IUD or implant removed before the method has expired, you feel comfortable removing it" and "You feel comfortable removing a client's IUD or implant at the client's request, even if you think she shouldn't have it removed". Answers were on a 5-point Likert scale ranging from "strongly disagree" to "strongly agree". We reported the proportion of providers who agreed with these statements, had been trained in either implant and/or IUD removals, and who provided removals in the past 12 months.

2.8 Analysis

a. Proportion of clients receiving desired LARC removals. We first estimated the proportion of clients who received a desired LARC removal. We then examined receipt of desired LARC removals by client characteristics. We used bivariable mixed-effects models that controlled for study arm of the facility in the initial Beyond Bias evaluation (intervention or control) and included a random intercept at the facility level to test the association between each characteristic and receipt of desired LARC removal.

b. Person-centered care by receipt of desired LARC removal. Among clients who were seeking a LARC removal and wanted to discontinue use or switch to another contraceptive method, we assessed whether person-centeredness of care varied by whether a client received a desired LARC removal. While we hypothesized that clients who did not receive their desired LARC removal during the visit would report having received less person-centered care, it is also possible that through conversation with the provider, the client decided to keep the LARC method and therefore reported receiving highly person-centered care. We fit a mixed-effects model to investigate how the PCFP autonomy and respect subscale varied by receipt of desired LARC removal, controlling for facility Beyond Bias intervention status, client age, marital status, parity, education, whether the client was accompanied to the visit, and perceived socioeconomic status. We also analyzed each measure included in the PCFP autonomy and respect subscale separately, comparing the distribution by receipt of LARC removal. Clients with missing outcome data were excluded from the analysis (<1%).

c. Provider training, provision, and attitudes. We estimated the proportion of providers who were trained in removal IUD and implants, when they reported receiving this training, and the proportion of providers who reported providing removals in the past year. We calculated the proportion of facilities where at least one provider reported providing LARC removals in the past year. We also summarized provider attitudes related to early LARC removals by examining the proportion of providers who agreed with the two statements and by looking at the mean across the Likert-style measures. We present findings for the pooled provider sample and for providers in the intervention and control arm separately. We also tested the bivariable association between provider training, provision of removals, and Beyond Bias intervention status and provider attitudes related to early LARC removals using mixed-effects models with random intercepts included by facility.

All data analyses were conducted in Stata v15 and R.

3. Results

i. Client sample description

Among 5,930 clients in Tanzania seeking a LARC removal, clients were on average 28 years old, and the majority were married (71%), had at least one child (98%), had completed less than secondary education (57%), and considered themselves in the middle socioeconomic category (61%) (Table 1).

ii. Receipt of a desired LARC method removal. Eighty-seven percent of clients received their desired LARC removal. Those who received the desired removal were more likely to have visited a facility enrolled in the Beyond Bias intervention (90% v 85%, p = .05), were 25 years or older (84% v 88%, p = .01), and were of the highest perceived socioeconomic status (94% of those who identified with the highest socioeconomic level had their method removed compared to 86% of those in the lowest, p = .01) (Table 1). Among the subset of clients who were asked about their pregnancy intentions (n = 5,452), clients who reported they wanted to get pregnant in the next six months were more likely to have received a desired LARC removal (98% received a removal) than every other group. Those who wanted to wait until they were married or finished with school were the least likely (89% had their LARC removed). Among implant users, 87% received their LARC removal compared to 81% among IUD users; however, there were relatively few IUD users in the sample (4% of clients who desired a LARC removal).

iii. Experiences of person-centered care. A total of 2,255 clients were included in our analysis of person-centered care. Among these clients, the PCFP autonomy and respect measure had a mean value of 2.58 and a standard deviation of .40 (possible range of 0–3, observed range: .7–3). Clients who received their desired LARC removal had higher scores on the PCFP autonomy and respect scale than clients who did not receive their desired LARC removal (Fig 1). We estimated those who received a desired LARC removal had scores that were, on average, .07 higher than those whose LARC was not removed (95% CI: .02 - .11, p = .006) (Fig 1B and S2 Table). Results differed according to whether the facility was enrolled in the Beyond Bias intervention (Fig 1B). While clients who received their desired LARC removal had higher scores on the PCFP autonomy and respect subscale across both treatment and control facilities (.20 in the control group and .26 in the treatment group) on average in the sample (Fig 1A), among control facilities, differences *between* facilities largely explained this difference—i.e., there was not a significant difference between clients by receipt of desired LARC removal within control facilities (β = .03, 95% CI: -.03 - .09, p = .38) in our mixed effects model. In treatment facilities, clients who had their LARC removed had PCFP scores that were .12 higher than among clients who did not get their LARC removed (95% CI: .05 - .20, p = .001).

Among individual measures of the PCFP respect and autonomy measure, 72% of those who had their LARC removed said the provider cared about them as a person all of the time, whereas only 37% of those who did not have their LARC removed responded this way (Table 2). Similar differences were observed for the other items: whether the respondent felt like they could trust the provider, whether the provider paid attention to them, and whether they could ask any questions. There were large differences in feeling involved in decisions about the care received by desired LARC removal receipt; however, in both groups many clients did not feel like they were always involved in decisions—39% of those who did not have their LARC removed felt they were always involved in decisions about their family planning choices compared to 57% among those who had their LARC removed.

Provider characteristics. Among the 259 providers surveyed, the majority were female, married, and parous, and were on average 40 years old with 5.5-year tenures at the facilities they worked (Table 3). The majority were trained as nurse or nurse-midwives (84%).

iv. Provider training and provision of LARC removals. Seventy-three percent of providers reported having received training on how to remove both IUDs and implants (Table 3). The majority of providers had received training on removals either in the last two years (25%-28%) or 3–5 years (43%-44%). Eighty-one percent of providers reported providing LARC removals in the past year (Table 3) and 97% of facilities had at least one provider that said they provided LARC removals. A larger proportion of providers in the intervention facilities reported providing method removals in the past year (87% v 77%, p = .1).

Table 1. Client characteristics and receipt of LARC removal in client exit survey *(n = 5,930)*.

	All clients seeking LARC removal N (%)	*Percent of clients who received LARC removal (n = 5,168) (%)*	*p-value*
All clients	*5,930* (100%)	87.2%	-
Beyond Bias intervention			
Control	3088 (52.1%)	84.5%	Ref
Treatment	2842 (47.9%)	90.0%	.05
Age (mean: 28, range: 15–55)			
≤19	153 (2.6%)	83.7%	Ref
20–24	1713 (28.9%)	86.5%	.12
25–29	1907 (32.2%)	87.1%	.03
30–34	1192 (20.1%)	88.0%	.01
35+	965 (16.3%)	87.9%	< .001
Relationship status			
Single	205 (3.5%)	91.2%	Ref
In a relationship, not living together	588 (9.9%)	87.2%	.16
In a relationship, living together	954 (16.1%)	85.7%	.13
Married	4181 (70.5%)	87.3%	.17
Parity (mean: 2, range: 0–8)			
No children	118 (2.0%)	83.1%	Ref
1 child	1959 (33.0%)	85.7%	.33
2 children	2011 (33.9%)	88.3%	.05
3+ children	1842 (31.1%)	87.7%	.06
Education			
Less than secondary	3369 (56.8%)	87.2%	Ref
Secondary or more	2561 (43.2%)	87.0%	.21
Perceived socioeconomic status*			
Lowest (Steps 1–2)	1920 (32.4%)	85.9%	Ref
Middle (Steps 3–4)	3623 (61.1%)	87.1%	.01
Highest (Steps 5–6)	387 (6.5%)	94.1%	.03
Accompanied to visit			
No	4149 (70.0%)	85.7%	Ref
Yes	1781 (30.0%)	90.5%	.52
Pregnancy intentions**			
Less than 6 months	553 (10.1%)	97.8%	Ref
Between 6 months and a year	431 (7.9%)	93.3%	< .01
Over a year and less than 5 years	2301 (42.2%)	91.7%	< .01
Between 5–10 years	1153 (21.1%)	92.5%	< .01
When I get married or after finish school	168 (3.1%)	88.7%	< .01
Do not want another child	846 (15.5%)	91.4%	< .01

First column shows sample proportions of clients by each characteristic. The second column displays the percent of clients who received their desired LARC removed within the strata of the given characteristic. P value estimated from mixed effects regressions controlling for facility intervention status and given variable with a random intercept on facility.

*Imagine six steps, where on the bottom, the first step, stand the poorest people, and on the highest step, the sixth, stand the rich.

** Asked only of a subset of clients who said they had received services at the facility that day (n = 5,474)

https://doi.org/10.1371/journal.pgph.0002810.t001

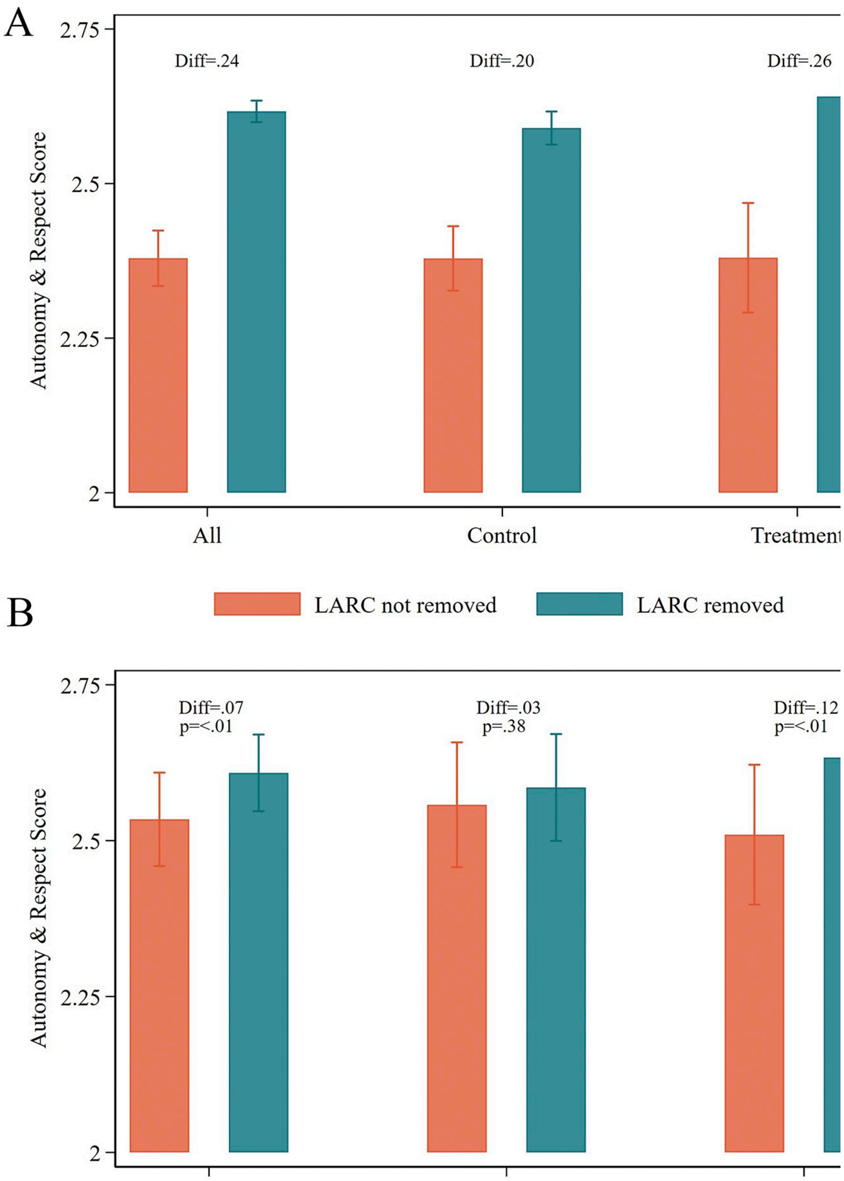

Fig 1. Respect and autonomy subscale of person-centered family planning scale by receipt of desired LARC removal among Tanzanian clients (A) Unadjusted averages observed in data (B) Modeled differences predicted through mixed effects model (N = 2,235). Modeled results estimated through mixed effects regressions with random effect on facility controlling for age, marital status, parity, education, socioeconomic status, and whether the client was accompanied to the facility. Receipt of desired LARC removal and facility intervention status were interacted. Unadjusted averages were calculated by taking the mean across all clients in the observed data. Bars represent 95% confidence intervals.

https://doi.org/10.1371/journal.pgph.0002810.g001

v. Provider attitudes on LARC removals. Only 51% of providers felt comfortable (answered 'strongly agree' or 'agree') removing a client's LARC method before it expired, and a similar proportion (49%) felt comfortable removing a client's LARC method even if they thought she should not have it removed. Providers in intervention facilities had more supportive attitudes on average; however, differences were not statistically significant (Table 3). Providers who reported providing removal services were more likely to express feeling uncomfortable removing LARCs in these two scenarios than those who said they did not provide removal services ($p < .05$).

Table 2. Table: Person centered family planning autonomy and respect subscale components by receipt of desired LARC removal among Tanzanian clients with LARC removal intention (N = 2,255).

	All		Treatment		Control	
	LARC Removed (n = 1,905)	LARC not removed (n = 350)	LARC Removed (n = 1,011)	LARC not removed (n = 91)	LARC Removed (n = 894)	LARC not removed (n = 259)
Not disrespected*	99.6	100.0	99.6	100.0	99.6	100.0
Not unfriendly*	96.0	94.9	97.7	90.1	94.0	96.5
Cared about you as person	71.8	37.4	70.9	41.8	72.8	35.9
Paid attention to you during stay	72.7	42.9	72.7	49.5	72.7	40.5
Completely trust provider	70.8	30.9	69.8	36.3	72.0	29.0
Involved you in decisions	56.9	38.6	58.9	41.8	54.7	37.5
Talked to you about how feeling	54.5	29.7	54.3	28.6	54.7	30.1
Felt they could ask any questions they had	65.5	37.7	64.5	42.9	66.7	35.9
Allowed to have someone stay during visit	93.3	96.9	95.4	94.5	90.9	97.7
Provider clearly explained things	63.9	45.3	64.5	54.4	63.3	42.1

Percentages represent the proportion of clients that answer the given question to indicate they were treated this way consistently in their visit. For positive statements the proportion represents the proportion of clients that answer "Yes, all the time" and for negative statements (denoted with a *) the proportion of clients that answer with "No, never".

https://doi.org/10.1371/journal.pgph.0002810.t002

4. Discussion

Our study found that while most clients received a LARC removal, 13% did not. Clients who did not receive their desired LARC removal were more likely to be of lower socioeconomic status and younger, and reported less autonomous and respectful care than clients who had their method removed. While the majority of providers reported being trained on LARC removals, only half felt comfortable removing the method at the client's request either before it had expired or when they thought the client was better off using the method.

Our findings indicating that older clients and socioeconomically advantaged clients seeking a LARC removal were more likely to get their method removed suggests that receipt of a desired LARC removal may be related to social status and power a client holds. Barriers that clients face to exercise their own reproductive autonomy may be stratified by social status, a finding echoed in literature around both LARC removal and promotion in the US [13, 14, 26–28]. There may be multiple causes of these underlying differences in receipt of desired LARC removal by client characteristics which may include structural factors (e.g., cost of removal services) [7], provider bias and discriminatory care based on client characteristics and beliefs about contraceptive methods [12, 13, 29], and/or that more resourced or older clients may have heightened feelings of agency and control making them better able to advocate for themselves [30].

Our findings indicate clients who did not receive their desired LARC removal received less autonomous and respectful care. In particular, our results point to disparities in how clients felt included in decision making and felt cared for during their visit. However, it is also important to note that overall differences in the PCFP scale in our study were relatively small. Previous research has documented how providers may enact barriers to removal for clients including preferring to treat side effects before removing the method, insisting on use when clients have many children or "short" birth intervals, emphasizing use of the method until its expiration, and judging the client's "readiness" for pregnancy [2, 7, 12, 31]. Providers' attitudes and willingness to remove a LARC appear to likely differ based on the client's stated reason for removal. Supporting this, we found that clients who did not intend to get pregnant in the next

Table 3. Provider characteristics and attitudes, training, and provision of LARC removals among providers in Tanzania (n = 259).

	All (n = 259)	Control (n = 141)	Treatment (n = 118)
Provider characteristics			
Age (mean) (SD)	39.9 (9.1)	39.3 (8.8)	40.6 (9.4)
Female	90.4%	91.5%	89.0%
Time working at facility (mean (SD))	5.5 (4.6)	5.1 (4.3)	6.1 (5.0)
Married	79.2%	79.9%	78.5%
Has children	90.7%	90.7%	90.7%
Cadre			
Doctor/Health officer	2.3%	.7%	5.1%
Nurse/nurse-midwife	84.2%	86.5%	81.4%
Midwife	13.1%	12.8%	13.6%
Training			
Trained on implant removals	84.9%	83.7%	86.4%
Trained on IUD removals	74.5%	74.5%	74.6%
Trained in both IUD and implant removals	73.0%	73.8%	72.0%
Implant removal training timing (n = 220)			
Last 1–2 years (2020–2021)	28.2%	26.3%	30.4%
Last 3–5 years (2017–2019)	44.1%	45.8%	42.2%
>5 years (2016 or before)	27.7%	28.0%	27.5%
IUD removal training timing (n = 193)			
Last 1–2 years (2020–2021)	25.4%	24.8%	26.1%
Last 3–5 years (2017–2019)	42.5%	44.8%	39.8%
>5 years (2016 or before)	32.1%	30.5%	34.1%
Reported providing LARC method removals in past year	81.4%	76.6%	87.3%
Attitudes			
Comfortable removing an IUD or implant before the method has expired (mean on scale of 1–5) (SD)	3.29 (1.02)	3.26 (1.04)	3.31 (1.01)
Comfortable removing a client's IUD or implant at the client's request, even if they think she shouldn't have it removed (mean on scale of 1–5) (SD)	3.19 (1.07)	3.12 (1.10)	3.28 (1.04)

https://doi.org/10.1371/journal.pgph.0002810.t003

year were less likely to receive a desired LARC removal and that providers had mixed comfort in removing LARCs before their expiration or at times they disagree with the decision the client has made. Providers' decisions to remove a LARC may be partially based on what a provider believes to be "appropriate" reproductive behavior for a client, what side effects they believe clients can tolerate, what contraceptive methods they think are appropriate for that client, and providers judgements about clients' pregnancy risk and 'readiness' [7, 31]. These judgements may in part be based on a clients' demographic traits and social position [28].

Clients who visited facilities that participated in the Beyond Bias intervention received LARC removals more often, suggesting an intervention to address bias with a focus on client choice and agency may work to address provider-imposed barriers to LARC removals. These changes were observed despite the Beyond Bias intervention not having an explicit focus on LARC removals. Future work should be designed to explicitly test whether the Beyond Bias intervention with added material on LARC removals, or similar interventions that include a larger focus on removals, address causes of refusal of LARC removals.

Within Beyond Bias intervention facilities, clients who did receive their desired LARC removal reported more autonomous and respectful care. However, within control facilities, there was little difference in the quality of care received between clients who received LARC

removal versus those who did not. As we did find differences in levels of person-centered care by receipt of LARC removal across control facilities, our findings suggest that in control facilities, facilities with lower levels of autonomous and respectful care were also the facilities that were least likely to provide LARC removals. One possible explanation for these findings is that the intervention created more interprovider variation. For instance, if a subset of providers who provided lower quality care and also less commonly removed LARCs did not change their behavior after the intervention, but other providers did—both improving in care quality and removing LARCs more frequently, then the intervention could have created a larger degree of association between person-centered care and LARC removal within facilities. Our findings in the control group indicate a potential need for targeted interventions for specific providers or facilities.

This study provides a detailed assessment of receipt of desired LARC removal and the person-centeredness of care among a large number of clients seeking a LARC removal. Despite this strength, there are several limitations to this study that should be mentioned. Past evidence demonstrates that patient-reported experiences with providers vary based on the reason presented for removal (expiration, side effects, partner discontent etc.) [7, 12]; however, the study instruments did not allow us to identify the reasons why clients wanted their method removed, how long they had been using the LARC, and for those who did not receive the LARC removal, why not. Moreover, facilities may lack necessary equipment or training on complicated removals, which may explain non-receipt of LARC removals. Second, while we aimed to capture client's intentions in their visit through the survey instrument questions, there may be misclassification in who is considered to have sought or received a LARC removal (e.g., there may be clients who we included in our analysis that were only visiting for advice about their LARC but did not want a removal that we could not capture using information in our survey). Because of the limitations in our survey tool, we only had information on pregnancy intention for a subset of our sample and therefore could not include this measure in our analysis of person-centered care. We do, however, look at differences in LARC removal receipt by pregnant intention where it is available. Finally, while we examined the association between receipt of a desired LARC removal and person-centered care, the results should not be interpreted causally since provider behavior and provider attitudes may confound the relationship.

5. Conclusion

This work adds to the growing body of literature on LARC removals in sub-Saharan Africa and suggests that while most clients are able to access a desired LARC removal, attention is needed to ensure that people are able to get a LARC removed when they want and that access to this service is equitable, a key component of high-quality autonomous contraceptive care and reproductive justice.

Supporting information

S1 Checklist. Inclusivity in global research.
(DOCX)

S1 Table. Items in autonomy and respect subscale of a validated *person-centered family planning scale*.
(DOCX)

S2 Table. Association between LARC removal and person-centered autonomous and respectful care: Model results (n = 2,235).
(DOCX)

Acknowledgments

We want to thank Zachary Wagner, the Principal Investigator on the Beyond Bias Evaluation. We thank Pathfinder International, especially Lydia Murithi, Mohamad Brooks, Xaher Gul, and Robert Kim for study support; and Muhammad Sharjeel, Sonia Surani, Madiha Latif Upendo Laizer, George Humba Wilfred Nyange, Eveline Kivuyo, Victor Dotto, Isihaka Mwandalima, Bakiono Bagnamboe, Daouda Son, Nestor Waongo, Pi´ezan Ganou, Sie Severin Kambire, Rodrigue Zagre, Bakary Mollogo, and Bruno Ki for implementing the intervention. We thank Rebecca de Guttry, Lynn Hu, Jean-Louis Koulidiati, and Muhammad Ali Awan for research support on the parent study.

Author Contributions

Conceptualization: Alexandra Wollum.

Data curation: Amon Sabasaba.

Formal analysis: Alexandra Wollum.

Methodology: Alexandra Wollum.

Supervision: Jessica D. Gipson.

Writing – original draft: Alexandra Wollum.

Writing – review & editing: Corrina Moucheraud, Amon Sabasaba, Jessica D. Gipson.

References

1. Jacobstein R. Liftoff: The blossoming of contraceptive implant use in Africa. Global Health: Science and Practice. 2018; 6: 17–39. https://doi.org/10.9745/GHSP-D-17-00396 PMID: 29559495
2. Brunie A, Aw FNRS, Ndiaye S, Dioh E, Lebetkin E, Lydon MM, et al. Making removals part of informed choice: a mixed-Method study of client experiences With removal of long-acting reversible contraceptives in Senegal. Global Health: Science and Practice. 2022 [cited 25 Sep 2022]. https://doi.org/10.9745/GHSP-D-22-00123 PMID: 36316132
3. Brandi K, Fuentes L. The history of tiered-effectiveness contraceptive counseling and the importance of patient-centered family planning care. Am J Obstet Gynecol. 2020; 222: S873–S877. https://doi.org/10.1016/j.ajog.2019.11.1271 PMID: 31794724
4. World Health Organization. Family Planning—A global handbook for providers. World Health Organization; 2018. Available: http://www.who.int/reproductivehealth/publications/fp-global-handbook/en/
5. Senderowicz L, Pearson E, Hackett K, Huber-Krum S, Francis JM, Ulenga N, et al. 'I haven't heard much about other methods': quality of care and person-centredness in a programme to promote the postpartum intrauterine device in Tanzania. BMJ Global Health. 2021; 6: e005775. https://doi.org/10.1136/bmjgh-2021-005775 PMID: 34162627
6. Christofield M, Lacoste M. Accessible contraceptive implant removal services: An essential element of quality service delivery and scale-up. Glob Health Sci Pract. 2016; 4: 366–372. https://doi.org/10.9745/GHSP-D-16-00096 PMID: 27577239
7. Callahan R, Lebetkin E, Brennan C, Kuffour E, Boateng A, Tagoe S, et al. What goes in must come out: A mixed-method study of access to contraceptive implant removal services in Ghana. Glob Health Sci Pract. 2020; 8: 220–238. https://doi.org/10.9745/GHSP-D-20-00013 PMID: 32606092
8. Senderowicz L. "I was obligated to accept": A qualitative exploration of contraceptive coercion. Social Science & Medicine. 2019; 239: 112531. https://doi.org/10.1016/j.socscimed.2019.112531 PMID: 31513932
9. Utaile MM, Debere MK, Nida ET, Boneya DJ, Ergano AT. A qualitative study on reasons for early removal of Implanon among users in Arba Minch town, Gamo Goffa zone, South Ethiopia: a phenomenological approach. BMC Women's Health. 2020; 20: 2. https://doi.org/10.1186/s12905-019-0876-1 PMID: 31896349
10. Yirgu R, Wood SN, Karp C, Tsui A, Moreau C. "You better use the safer one. . . leave this one": the role of health providers in women's pursuit of their preferred family planning methods. BMC Women's Health. 2020; 20: 170. https://doi.org/10.1186/s12905-020-01034-1 PMID: 32787924

11. Brunie A, Lydon MM, Ndiaye S, Aw FNRS, Lebetkin E, Cartwright A, et al. Ensuring sufficient service capacity for removals of long-acting reversible contraceptives: a mixed-method study of provider experiences in Senegal. Gates Open Res. 2022; 6: 46. https://doi.org/10.12688/gatesopenres.13600.1 PMID: 35919828
12. Senderowicz L, Kolenda A. "She told me no, that you cannot change": Understanding provider refusal to remove contraceptive implants. SSM—Qualitative Research in Health. 2022; 2: 100154. https://doi.org/10.1016/j.ssmqr.2022.100154 PMID: 37304900
13. Higgins JA, Kramer RD, Ryder KM. Provider bias in long-acting reversible contraception (LARC) promotion and removal: Perceptions of young adult women. Am J Public Health. 2016; 106: 1932–1937. https://doi.org/10.2105/AJPH.2016.303393 PMID: 27631741
14. Manzer JL, Bell AV. The limitations of patient-centered care: The case of early long-acting reversible contraception (LARC) removal. Social Science & Medicine. 2022; 292: 114632. https://doi.org/10.1016/j.socscimed.2021.114632 PMID: 34891032
15. NWHN-SisterSong Joint statement of principles on LARCs. [cited 2 Feb 2023]. Available: https://nwhn.org/nwhn-joins-statement-principles-larcs/
16. World Health Organization. Ensuring human rights in the provision of contraceptive information and services. World Health Organization; 2014. Available: http://www.who.int/reproductivehealth/publications/family_planning/human-rights-contraception/en/
17. American College of Obstetricians and Gynecologists. Patient-centered contraceptive counseling. Committee Statement. 2022;No. 1. Available: https://www.acog.org/en/clinical/clinical-guidance/committee-statement/articles/2022/02/patient-centered-contraceptive-counseling
18. Ross L, Derkas E, Peoples W, Roberts L, Bridgewater P. Radical reproductive justice: Foundation, theory, practice, critique. Feminist Press at CUNY; 2017.
19. Sudhinaraset M, Afulani PA, Diamond-Smith N, Golub G, Srivastava A. Development of a person-centered family planning scale in India and Kenya. Studies in Family Planning. 2018; 49: 237–258. https://doi.org/10.1111/sifp.12069 PMID: 30069983
20. Institute of Medicine. Crossing the Quality Chasm: A new health system for the 21st century. Washington, DC: The National Academies Press; 2001. https://doi.org/10.17226/10027
21. Sergison JE, Stalter RM, Callahan RL, Rademacher KH, Steiner MJ. Cost of contraceptive implant removal services must be considered when responding to the growing demand for removals. Global Health: Science and Practice. 2017; 5: 330–332. https://doi.org/10.9745/GHSP-D-17-00100 PMID: 28655806
22. Ministry of Health, Community Development, Gender, Elderly and Children. Tanzania national family planning costed implementation plan: 2019–2023. 2019. Available: https://fp2030.org/sites/default/files/Tanzania_CIP_2019-2023.pdf
23. Wagner Z, Moucheraud C, Wollum A, Friedman W, Shah M, Dow W. Addressing provider bias in contraceptive service delivery for youth and adolescents: An Evaluation of the Beyond Bias Project. 2022 Mar. Available: https://www.pathfinder.org/wp-content/uploads/2022/03/Beyond-Bias-Project-Evaluation-Brief-English.pdf
24. Murithi L, Gibbs T, Hope R. Integrating human-centered design in a multidisciplinary effort to address provider bias: A summary of the Beyond Bias experience. 2021 Jul. Available: https://hcdexchange.org/resource-repository/integrating-human-centered-design-in-a-multidisciplinary-effort-to-address-provider-bias-the-beyond-bias-experience/
25. Howe LD, Hargreaves JR, Ploubidis GB, De Stavola BL, Huttly SRA. Subjective measures of socio-economic position and the wealth index: a comparative analysis. Health Policy and Planning. 2011; 26: 223–232. https://doi.org/10.1093/heapol/czq043 PMID: 20817696
26. Dehlendorf C, Ruskin R, Grumbach K, Vittinghoff E, Bibbins-Domingo K, Schillinger D, et al. Recommendations for intrauterine contraception: a randomized trial of the effects of patients' race/ethnicity and socioeconomic status. American Journal of Obstetrics and Gynecology. 2010; 203: 319.e1–319.e8. https://doi.org/10.1016/j.ajog.2010.05.009 PMID: 20598282
27. Gomez AM, Fuentes L, Allina A. Women or LARC first? Reproductive autonomy and the promotion of long-acting reversible contraceptive methods. Perspect Sex Reprod Health. 2014; 46: 171–175. https://doi.org/10.1363/46e1614 PMID: 24861029
28. Manzer JL, Bell AV. "We're a Little Biased": Medicine and the management of bias through the case of contraception. J Health Soc Behav. 2021; 62: 120–135. https://doi.org/10.1177/00221465211003232 PMID: 33843323
29. Solo J, Festin M. Provider bias in family planning services: A review of its meaning and manifestations. Global Health: Science and Practice. 2019; 7: 371–385. https://doi.org/10.9745/GHSP-D-19-00130 PMID: 31515240

30. Afulani PA, Ogolla BA, Oboke EN, Ongeri L, Weiss SJ, Lyndon A, et al. Understanding disparities in person-centred maternity care: the potential role of provider implicit and explicit bias. Health Policy and Planning. 2021; 36: 298–311. https://doi.org/10.1093/heapol/czaa190 PMID: 33491086

31. Amico JR, Bennett AH, Karasz A, Gold M. "I wish they could hold on a little longer": physicians' experiences with requests for early IUD removal. Contraception. 2017; 96: 106–110. https://doi.org/10.1016/j.contraception.2017.05.007 PMID: 28578147
